# Inflammatory Biomarkers in Acute Suicidal Behaviors

**DOI:** 10.3390/ijms27041691

**Published:** 2026-02-09

**Authors:** Magdalena Lewandowska, Jakub Leszczyński-Czeczatka, Mariusz Siemiński

**Affiliations:** 1 University Clinical Centre, Emergency Department, Medical University of Gdansk, 80-214 Gdansk, Poland; lewandowska.magdalena@gumed.edu.pl (M.L.); jczeczatka@gmail.com (J.L.-C.); 2 Department of Emergency Medicine, Medical University of Gdansk, 80-214 Gdansk, Poland

**Keywords:** biomarkers, suicide, depression, interleukins, inflammatory, acute phase proteins

## Abstract

This review focuses on suicidal ideation and attempts in the context of major depressive disorder. Despite clinical advances, suicide risk assessment still relies mainly on subjective evaluation. Emerging evidence highlights immune-inflammatory dysregulation as a biological link between depression and suicidality. This review summarizes current findings on inflammatory biomarkers as potential predictors of suicidal behavior. The discussed markers include acute phase proteins (C-reactive protein, homocysteine), hematological indices from routine blood tests (NLR, PLR, MLR, SII, NAR), and cytokines (IL-6, TNF-α, IL-1β, IL-4, IL-10). Many studies report increased levels of CRP, IL-6, TNF-α, and IL-1β and decreased IL-4 and IL-10 in suicidal individuals, reflecting an imbalance between the immune-inflammatory response system (IRS) and the compensatory immune-regulatory reflex system (CIRS). Such dysregulation may promote neuroinflammation and neurotoxicity via the kynurenine pathway. Hematological ratios offer inexpensive, accessible indicators that could complement psychiatric evaluation. However, heterogeneity across studies, lack of standardized cut-off values, and the influence of confounders such as age, sex, and medication limit their clinical utility. Inflammatory biomarkers currently hold potential as objective adjuncts—but not substitutes—to clinical judgment in assessing suicide risk.

## 1. Introduction

### 1.1. Depression—Epidemiology

Depression is the most common mental disorder associated with suicidal risk. According to the World Health Organization (WHO), over 300 million people worldwide suffer from depression, representing one of the major contributors to the global burden of disease [1]. This condition significantly impairs social and occupational functioning, leading to disability and reduced quality of life. Depression affects nearly all aspects of an individual’s daily functioning—from emotional and cognitive domains to social and physical well-being—which is reflected in high disability-adjusted life years (DALYs) [2]. Comorbid somatic and psychiatric disorders further exacerbate the functional and occupational burden of major depressive disorder [3]. It is estimated that up to 90% of individuals who die by suicide have a prior mental diagnosis, with major depressive disorder being the most prevalent [4]. Although the relationship between depression and suicidal behavior is well established, objective methods for identifying individuals at highest risk remain insufficient [5]. In this review, we focus specifically on suicidal ideation and suicide attempts occurring during major depressive disorder (MDD). Although suicidality is also prevalent in other psychiatric conditions (e.g., bipolar disorder or psychotic disorders), a cross-diagnostic comparison of biomarker profiles is beyond the scope of this manuscript and is discussed as an important direction for future research. Although the primary focus of this review is suicidality in major depressive disorder, several high-quality meta-analyses include mixed affective populations; their findings are therefore interpreted cautiously as supportive, but not disorder-specific, evidence.

### 1.2. Suicide Attempts—Epidemiology

Suicide accounts for over 727,000 deaths annually, making it the third leading cause of death among individuals aged 15–29 years [6]. Non-fatal suicide attempts substantially outnumber completed suicides in many populations, highlighting the magnitude of the problem [7]. A previous suicide attempt is one of the strongest predictors of future suicidal behavior—individuals with such history have several-fold higher risk of recurrence [8]. The frequency of suicide attempts varies depending on age, sex, and geographical region; nevertheless, they consistently represent a major burden to healthcare systems and families worldwide [9]. In this review, we address suicidal ideation and suicide attempts within depressive disorders, particularly MDD, as the target clinical context for biomarker interpretation.

### 1.3. Psychiatric Assessment and the Need for Objective Biomarkers

Clinical assessment of suicide risk primarily relies on subjective evaluation and patient self-report. Accordingly, the biomarker literature discussed below is interpreted primarily in the context of MDD-related suicidality in acute-care settings. Although screening tools such as the SAD PERSONS scale are commonly used in emergency departments, their predictive value remains limited—characterized by high specificity but consistently low sensitivity—and therefore should not be used as standalone decision tools [10]. Psychiatrists are not always available in acute care settings, and critical decisions often fall on physicians of other specialties. Current clinical prediction models for suicide attempts and deaths have limited accuracy, restricting reliable risk assessment. Improving prediction requires the incorporation of additional clinically meaningful parameters [11].

### 1.4. Inflammatory Biomarkers and Suicidality

This review focuses primarily on inflammatory biomarkers. Metabolic markers are discussed only when they are mechanistically linked to inflammatory or immune-mediated pathways relevant to suicidal behavior. An increasing body of evidence, including recent meta-analytic data, supports the role of immune-inflammatory mechanisms in the pathophysiology of both depression and suicidal behavior [12,13]. Nonspecific hematological indices such as the neutrophil-to-lymphocyte ratio (NLR), monocyte-to-lymphocyte ratio (MLR), and platelet-to-lymphocyte ratio (PLR) are gaining interest as easily accessible and inexpensive inflammatory markers [14]. Similarly, C-reactive protein (CRP), a commonly used clinical biomarker, has been associated with the risk of suicide attempts and suicidal behavior [15]. Proinflammatory cytokines—including interleukin-6 (IL-6), interleukin-1β (IL-1β), and tumor necrosis factor-alpha (TNF-α)—along with anti-inflammatory cytokine IL-10, may also reflect suicidal risk through dysregulation of immune homeostasis [16]. Additional relevant biomarkers include homocysteine and various oxidative stress indicators, both of which have been linked to neuroinflammation and neurotoxicity [17,18]. Epidemiological findings on infectious exposure and suicidality are mixed: some studies and meta-analyses suggest associations for specific pathogens (e.g., T. gondii), whereas other longitudinal population studies report no clear association for common infections. These results underscore potential confounding (including infection-related inflammation) when interpreting peripheral inflammatory biomarkers [19,20].

### 1.5. Aim of the Review

The aim of this narrative review is to synthesize current evidence on peripheral inflammatory biomarkers associated with suicidal ideation and suicide attempts in major depressive disorder (MDD). We focus on clinically accessible markers—acute-phase proteins (e.g., C-reactive protein, homocysteine), hematological indices derived from routine complete blood count, and selected cytokines—to evaluate their potential role as adjuncts to clinical suicide risk assessment. We also summarize proposed biological mechanisms linking peripheral immune activation with neuroinflammatory processes relevant to suicidality, and we discuss methodological limitations, confounders, and priorities for future research.

**Figure 1:**
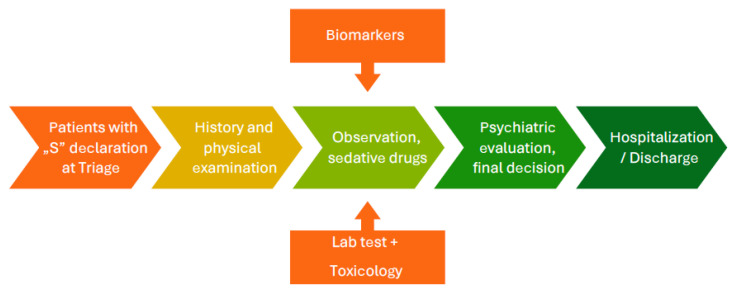
Place of biomarkers of suicidal behaviors in process of emergency department.

Biochemical assessment using accessible inflammatory biomarkers could be of particular value in emergency settings as an adjunct to clinical evaluation, potentially contributing additional objective information to the assessment of suicidal risk, rather than serving as standalone diagnostic tools. We propose a place for such biomarkers in process of emergency department on Figure 1.

## 2. Methods

### 2.1. Literature Search Strategy

A comprehensive literature search was conducted to identify peer-reviewed studies examining inflammatory biomarkers, including selected hematological indices and metabolically related markers linked to immune-inflammatory pathways associated with suicidal behavior. Searches were performed in PubMed, Scopus, and Web of Science between January 2000 and December 2024. Additional manual screening of the reference lists of relevant reviews and meta-analyses was undertaken to ensure coverage of seminal publications. The initial database search identified 522 records. After removal of duplicates, 343 unique articles remained. Following title and abstract screening, 76 full-text articles were assessed for eligibility, of which 43 were included in the final qualitative synthesis.

### 2.2. Definitions of Suicidal Behaviors

Definitions of suicidal behaviors used in this review are based on the standardized nomenclature proposed by Silverman et al. [21,22]. Suicidal ideation refers to thoughts of engaging in behavior intended to end one’s life, without execution of the act. Suicide attempt is defined as a self-injurious behavior with at least some intent to die, which does not result in death. Suicide death refers to a fatal self-inflicted act with evidence of intent to die. In the present review, the analysis is limited to suicidal ideation and suicide attempts occurring in the context of major depressive disorder; suicide death was not systematically analyzed due to heterogeneity of available data.

### 2.3. Eligibility Criteria

Studies were eligible for inclusion if they investigated inflammatory biomarkers in individuals presenting with suicidal ideation or suicide attempts in the context of depressive disorders, particularly major depressive disorder. Eligible biomarkers included acute-phase proteins, hematological indices derived from routine blood tests, and cytokines related to immune-inflammatory pathways. Both observational studies (cross-sectional, case–control, cohort) and evidence syntheses (systematic reviews and meta-analyses) published in peer-reviewed journals were considered.

Studies were excluded if they were case reports or case series with fewer than 10 participants, non-human studies, conference abstracts, editorials without original data, or articles lacking clearly defined biomarker assessments. Studies focusing exclusively on suicide death or on primary psychotic or bipolar disorders without separate analysis of depressive populations were also excluded. The term “related biological alterations” refers to peripheral inflammatory changes associated with immune activation, oxidative stress, or neurotoxic pathways relevant to suicidal behavior.

## 3. Results

### 3.1. Overview and Interpretative Framework

The available literature on inflammatory biomarkers in suicidality is highly heterogeneous with respect to study design, clinical populations, and outcome definitions. Most studies compare patients with depressive disorders and suicidal ideation or suicide attempts to either healthy controls or non-suicidal psychiatric controls, while direct comparisons across all three groups (healthy individuals, depressed patients without suicidality, and depressed patients with suicidality) are relatively scarce. Moreover, most studies focus on suicidal ideation or attempts rather than suicide death. Consequently, the present review provides a narrative synthesis of group-level associations rather than a systematic comparative analysis.

### 3.2. Main Groups of Biomarkers

This review focuses on suicidal ideation and suicide attempts; suicide death was not systematically analyzed due to limited and heterogeneous data. The search for objective biomarkers of suicidal risk has led to increasing interest in inflammatory markers measurable in serum or peripheral blood. Several groups of parameters have been proposed, ranging from acute phase proteins to hematological indices and specific cytokines. These biomarkers are relatively easy to obtain in routine laboratory testing and may complement psychiatric assessment by providing additional objective data.

Acute phase proteins, such as C-reactive protein (CRP) and homocysteine, are among the most frequently studied indicators. Elevated CRP levels have been associated with suicide attempts and with depressive symptom burden in some clinical samples, although the strength of this relationship varies across studies [16,23,24]. Homocysteine, a metabolic marker linked to impaired methylation processes and oxidative stress, has also been investigated as a potential correlate of suicidality, with preliminary studies suggesting an association with suicide attempts [17].

Hematological and biochemical indices represent another group of promising biomarkers. Ratios derived from complete blood count, including the neutrophil-to-lymphocyte ratio (NLR), platelet-to-lymphocyte ratio (PLR), and monocyte-to-lymphocyte ratio (MLR), have been frequently associated with depression and suicidal behavior [12,25]. Other indices, such as the neutrophil-to-albumin ratio (NAR) or systemic immune-inflammation index (SII), have been less frequently studied but may reflect a broader proinflammatory state contributing to psychiatric vulnerability. Their clinical advantage lies in the fact that they are inexpensive and widely available, although methodological heterogeneity across studies limits their immediate applicability.

Proinflammatory cytokines constitute the third major category. Increased levels of interleukin-6 (IL-6), tumor necrosis factor alpha (TNF-α), and interleukin-1β (IL-1β) have been observed in patients with depression and in individuals after suicide attempts, supporting the hypothesis of an immune-inflammatory contribution to suicidality [13,16]. Conversely, anti-inflammatory cytokines such as interleukin-4 (IL-4) and interleukin-10 (IL-10) may exert regulatory or protective effects through activation of the compensatory immune-regulatory reflex system (CIRS), which counterbalances the immune-inflammatory response system (IRS). However, findings regarding these cytokines remain inconsistent, as some studies report reduced IL-4 and IL-10 levels in patients with suicidal behavior, while others observe no significant differences compared to non-suicidal psychiatric controls. Meta-analytic evidence indicates that suicidal behavior is associated with increased inflammatory and neurotoxic activity and reduced neuroprotective markers [13]. Clinical data further support an imbalance between IRS and CIRS functioning, reflected in elevated proinflammatory and reduced anti-inflammatory cytokines in individuals with suicidality [26].

Taken together, these three groups of biomarkers—acute phase proteins, hematological indices, and cytokines—provide converging evidence that inflammatory mechanisms are linked to suicidal behavior. Nonetheless, their predictive value remains limited at the individual level, emphasizing the need for further multicenter, prospective studies with standardized sampling and analytical protocols.

### 3.3. Acute Phase Proteins

Acute phase proteins represent one of the earliest indicators of systemic inflammatory activation and are increasingly recognized as potential correlates of suicidal behavior. Among these proteins, C-reactive protein (CRP) and homocysteine have received the most attention in psychiatric research due to their clinical accessibility and established link with neuroinflammatory and oxidative stress pathways.

#### 3.3.1. C-Reactive Protein (CRP)

CRP is an acute-phase reactant synthesized by hepatocytes under the stimulation of proinflammatory cytokines such as interleukin-6 (IL-6) and tumor necrosis factor-alpha (TNF-α). Elevated CRP concentrations have been frequently associated with major depressive disorder (MDD), particularly in patients exhibiting suicidal ideation or a history of suicide attempts.

Evidence from both meta-analyses and clinical studies demonstrates a moderate but significant elevation of CRP levels in suicidal individuals compared with non-suicidal psychiatric and healthy controls [23,24]. For instance, Miola et al. (2021) reported a standardized mean difference (SMD) of 0.39 (95% CI: 0.23–0.55), suggesting a systemic inflammatory state linked to suicidal behavior [23]. However, results remain heterogeneous, partly due to variations in assay methods, timing of sampling relative to suicidal events, and clinical confounders such as body mass index, infection, or metabolic comorbidities [24].

Although the meta-analytic evidence supporting CRP elevation in suicidal behavior is based on relatively large and methodologically robust datasets—including Miola et al.’s [23] pooled analysis of over 4000 participants—the overall certainty of findings remains moderate due to substantial heterogeneity across studies, variability in clinical populations, and the strong influence of confounding metabolic and inflammatory factors.

#### 3.3.2. Homocysteine

Homocysteine is a sulfur-containing amino acid formed during methionine metabolism. Elevated serum homocysteine (hyperhomocysteinemia) has been linked to endothelial dysfunction, oxidative stress, and excitotoxicity—all of which may contribute to neurobiological vulnerability in depression and suicidal behavior [27,28].

Preliminary evidence suggests that higher homocysteine levels are present in patients with suicide attempts compared with non-suicidal depressed individuals [17]. These findings have led to the hypothesis that disruptions in one-carbon metabolism—particularly deficiencies in folate, vitamin B6, or B12—may contribute to neuroinflammation and impaired monoaminergic neurotransmission [29,30].

Nevertheless, current data remain inconclusive; some studies report no significant difference in homocysteine levels between suicidal and non-suicidal depressed patients. For example, in a recent clinical cohort study, no significant association between serum homocysteine concentrations and suicide attempts was observed in major depressive populations, highlighting inconsistency across samples and methodologies [31]. This inconsistency underscores the need for standardized methodologies and large-scale, longitudinal designs assessing temporal dynamics of homocysteine in relation to suicidal risk.

Although preliminary studies—generally based on modest sample sizes—suggest a potential association between elevated homocysteine and suicide attempts, the overall evidence remains weak due to inconsistent findings across cohorts and the absence of large, well-controlled longitudinal studies capable of clarifying whether homocysteine represents a causal risk marker or merely reflects metabolic and nutritional confounders. In line with these findings, earlier longitudinal studies in depressive populations have shown that homocysteine and related one-carbon metabolism markers exhibit limited predictive value when considered in isolation, underscoring the restricted clinical utility of single metabolic biomarkers [30].

#### 3.3.3. Interpretation and Limitations

While both CRP and homocysteine are promising as peripheral inflammatory markers, their diagnostic or prognostic value in individual suicide risk assessment is currently limited. They may serve as complementary biomarkers reflecting the broader inflammatory milieu, particularly when interpreted alongside hematological indices (e.g., NLR, PLR) or cytokine panels (e.g., IL-6, TNF-α).

Future multicenter studies employing multimodal approaches—combining clinical, biochemical, and neuroimaging parameters—are essential to clarify the mechanistic and temporal relationship between acute-phase proteins and suicidal behavior. Recent clinical studies further suggest that no single inflammatory biomarker is sufficient to characterize suicidal risk, and that combined or integrative biomarker approaches may better reflect the complex immune dysregulation observed in major depressive disorder with suicidality [13,26].

### 3.4. Hematological Inflammatory Indices Derived from Complete Blood Count

This review focuses on suicidal ideation and suicide attempts; suicide death was not systematically analyzed due to limited and heterogeneous data. Several inflammatory indices derived from routine complete blood count (CBC) have been proposed as indirect markers of systemic inflammation. These include the neutrophil-to-lymphocyte ratio (NLR), platelet-to-lymphocyte ratio (PLR), and monocyte-to-lymphocyte ratio (MLR), which reflect the balance between innate immune activation and adaptive immune regulation. Peripheral hematological indices derived from routine complete blood count (CBC) have emerged as easily obtainable and cost-effective markers of systemic inflammation. These indices—particularly the neutrophil-to-lymphocyte ratio (NLR), platelet-to-lymphocyte ratio (PLR), monocyte-to-lymphocyte ratio (MLR), and derived composite markers such as the systemic immune-inflammation index (SII) and neutrophil-to-albumin ratio (NAR)—have attracted increasing attention as potential correlates of depression and suicidal behavior.

#### 3.4.1. Neutrophil-to-Lymphocyte Ratio (NLR)

NLR is a widely studied index reflecting the balance between innate (neutrophil-mediated) and adaptive (lymphocyte-mediated) immune responses. Elevated NLR values indicate a proinflammatory shift and relative immunosuppression, mechanisms thought to contribute to the neuroinflammatory model of suicidal behavior.

Elevated NLR has been frequently reported across mood disorders [32], and systematic evidence indicates that NLR is further increased in patients exhibiting suicidal behavior compared with both non-suicidal depressed individuals and healthy controls [14]. Kılıç et al. (2024) [12] additionally demonstrated that higher NLR distinguishes suicide attempters from non-attempters with major depressive disorder, independent of age, sex, or antidepressant use [12]. These findings support the hypothesis that low-grade systemic inflammation may contribute to suicidal vulnerability. Although meta-analytic and clinical data frequently show elevated NLR in individuals with suicidal behavior, the strength of this evidence is limited by the predominantly cross-sectional design of available studies and relatively small sample sizes in key clinical cohorts—such as Kılıç et al. (2024) [12]—highlighting the need for larger, prospectively designed investigations to confirm NLR’s reliability as a biomarker of suicidal vulnerability. Across available studies, NLR is generally higher in depressed patients with suicidal ideation or suicide attempts compared to healthy controls. Fewer studies directly compare suicidal and non-suicidal depressed patients; however, those that do suggest higher NLR values in suicidal subgroups. Due to heterogeneity in study design and sample characteristics, consistent effect sizes and clinically applicable cut-off values cannot be established.

#### 3.4.2. Platelet-to-Lymphocyte Ratio (PLR) and Monocyte-to-Lymphocyte Ratio (MLR)

Both PLR and MLR have been investigated as supplementary indices of inflammatory activation. Alterations in platelet parameters have been described in depressive and anxiety disorders [31], and dysregulation of the platelet serotonergic system has been linked to suicidal behavior [32,33], suggesting that platelet-related mechanisms may contribute to affective dysregulation even though direct evidence for PLR in suicidality remains limited. Similarly, MLR elevation can indicate monocyte-driven cytokine release and activation of microglia-related pathways.

A systematic review by Velasco et al. (2023) analyzed 11 studies and found preliminary evidence of elevated NLR in depressed patients with suicidal behavior; the findings for PLR and MLR were suggestive but still under-investigated [14]. However, heterogeneity in cutoff values and the cross-sectional nature of most studies limit the generalizability of these findings.

Given that most studies evaluating PLR and MLR are small, cross-sectional, and often secondary analyses within broader inflammatory profiles, the current evidence base—summarized in Velasco et al.’s [14] review of 11 heterogeneous studies—remains insufficient to establish these indices as reliable biomarkers of suicidality, underscoring the need for larger, methodologically standardized investigations. Evidence for PLR and MLR is more limited and inconsistent. While some studies report elevations in suicidal compared to non-suicidal depressed patients, others show no significant differences, highlighting the exploratory nature of these indices.

#### 3.4.3. Composite Indices (SII, NAR, Others)

Emerging hematological indices such as the systemic immune-inflammation index (SII = platelets × neutrophils/lymphocytes) and the neutrophil-to-albumin ratio (NAR) have been explored as integrative markers of systemic inflammatory status, combining cellular and biochemical parameters [34,35,36].

Preliminary findings suggest that SII may be elevated in individuals with suicidal behavior; however, available studies are small and cross-sectional, and evidence for NAR remains scarce and largely exploratory, with most research focusing on mood disorders rather than suicidality [34,35,36]. To date, no validated reference ranges or cut-off values exist for SII or NAR in psychiatric populations, and studies remain small and single-center. Nonetheless, the current evidence is limited to small, single-center samples, and no validated cut-off values are yet available for clinical application.

The study by Ninla-Aesong et al. (2024) [37] is based on relatively small, single-center samples, which limits the robustness and generalizability of their findings. Evidence regarding SII and especially NAR remains exploratory, with most research focusing on mood disorders rather than suicidality. The lack of standardized reference ranges, coupled with methodological heterogeneity across studies, further constrains the clinical interpretability of these biomarkers.

#### 3.4.4. Interpretation

Taken together, hematological indices constitute a promising and pragmatic toolset for quantifying peripheral inflammation in psychiatric populations. Their main advantage lies in accessibility, low cost, and reproducibility in standard clinical laboratories.

However, these biomarkers are relatively nonspecific and their levels may be affected by a range of methodological and clinical confounders—such as differences in sampling time (diurnal variation), lack of control for BMI (Body Mass Index), smoking status or medication use—which limits current clinical translation [13]. Therefore, their interpretation in suicide risk assessment should remain cautious and context-dependent, ideally integrated with cytokine and clinical data rather than used as standalone predictors. Direct comparisons of inflammatory indices across diagnostic categories (e.g., major depressive disorder, bipolar disorder, or psychotic disorders) in suicidal populations are scarce. Therefore, the present review does not allow conclusions regarding diagnosis-specific biomarker profiles, underscoring the need for future transdiagnostic studies.

### 3.5. Proinflammatory Cytokines

Cytokines are key mediators of immune communication that regulate inflammatory signaling between the peripheral and central nervous systems. Dysregulation of cytokine balance—characterized by excessive activation of proinflammatory pathways and insufficient compensatory anti-inflammatory responses—has been generally implicated in the pathophysiology of major depressive disorder (MDD) and suicidal behavior. This concept aligns with the immune-inflammatory response system (IRS) and compensatory immune-regulatory reflex system (CIRS) framework proposed by Maes and colleagues, describing disturbed homeostatic feedback between pro- and anti-inflammatory circuits [38]. This conceptual framework is supported by translational models of immune dysregulation in depression, while meta-analytic evidence further implicates immune-inflammatory and neurotoxic pathways in suicidal behavior, although underlying studies vary in size and methodological consistency [13,38].

#### 3.5.1. Proinflammatory Cytokines: IL-6, TNF-α, and IL-1β

Among proinflammatory mediators, interleukin-6 (IL-6), tumor necrosis factor-alpha (TNF-α), and interleukin-1β (IL-1β) are the most often studied cytokines in affective disorders. These mediators are implicated in neuroinflammatory processes and have been linked to alterations in immune–brain communication relevant to mood dysregulation and suicidality [39].

Meta-analyses have demonstrated elevated serum IL-6 and TNF-α concentrations in suicidal individuals compared to non-suicidal psychiatric controls and healthy populations [13,39]. Post-mortem findings are discussed here exclusively to support mechanistic plausibility and are not considered directly comparable to peripheral biomarkers assessed in living patients. Although IL-6 levels were increased in individuals with suicide attempts, their weak or inconsistent correlations with clinical severity measures indicates that IL-6 cannot yet be considered a trait marker of suicidal behavior [16].

Similarly, inflammatory signaling—including IL-1β—has been linked to suicidal behavior; meta-analytic evidence indicates group-level elevations of IL-1β in suicidality [40], while post-mortem studies in severe depression show increased quinolinic acid in cortical regions, consistent with kynurenine-pathway-related neurotoxicity [24]. These findings collectively support the hypothesis that persistent low-grade inflammation may induce neurotoxic cascades and impair neuroplasticity within corticolimbic circuits involved in mood and impulse control.

The cited meta-analyses and post-mortem studies provide robust group-level evidence for elevations of IL-6, TNF-α, and IL-1β in suicidality, although sample heterogeneity and weak associations with clinical severity limit their interpretive specificity.

#### 3.5.2. Anti-Inflammatory Cytokines: IL-4 and IL-10

In contrast, interleukin-4 (IL-4) and interleukin-10 (IL-10) represent anti-inflammatory cytokines that mitigate excessive immune activation by suppressing macrophage and microglial responses. While several studies have reported reduced IL-4 and IL-10 levels in suicide attempters, the findings are inconsistent and do not conclusively demonstrate CIRS dysfunction in the context of IRS hyperactivity [13,35]. However, other studies have reported inconsistent or nonsignificant findings, underscoring the heterogeneity of immune responses and the potential influence of clinical comorbidities, medication exposure, and methodological factors.

This inconsistency likely reflects the dynamic and context-dependent nature of cytokine release, which may vary across acute, chronic, or treatment-modulated stages of depression.

Current evidence on IL-4 and IL-10 in suicidality is inconsistent, as most studies involve small, heterogeneous samples and yield mixed or nonsignificant results, limiting any firm conclusions about anti-inflammatory (CIRS) dysfunction in suicidal behavior.

#### 3.5.3. Cytokine Networks and Neuroinflammatory Mechanisms

In the context of depressive disorders and suicidality, the term “neurotoxicity” refers to inflammation-driven neurobiological processes rather than direct neuronal cell death. Specifically, peripheral proinflammatory cytokines may activate indoleamine-2,3-dioxygenase (IDO), shifting tryptophan metabolism toward the kynurenine pathway and increasing the production of neuroactive metabolites such as quinolinic acid. Quinolinic acid acts as an NMDA receptor agonist, contributing to glutamatergic excitotoxicity, microglial activation, impaired neuroplasticity, and dysfunction of corticolimbic circuits involved in mood regulation and impulse control. Peripheral inflammatory signals may influence central nervous system function through several mechanisms, including cytokine transport across the blood–brain barrier, activation of endothelial and microglial cells, and vagal or humoral immune–brain signaling. These pathways allow peripheral immune activation to translate into central neuroinflammatory and neurotoxic processes relevant to suicidal behavior.

Recent research indicates that suicidal behavior is not driven by a single cytokine abnormality but by a network-level dysregulation involving multiple inflammatory mediators, oxidative stress pathways, and tryptophan–kynurenine metabolism [24,35,41].

Proinflammatory cytokines can activate indoleamine-2,3-dioxygenase (IDO), thereby shifting tryptophan metabolism toward the kynurenine pathway and increasing the production of neuroactive metabolites such as quinolinic acid, an NMDA receptor agonist that contributes to glutamatergic excitotoxicity [40,42].

This mechanistic pathway provides a plausible biological link between peripheral inflammation and central neurotoxicity observed in suicidal behavior.

Current evidence suggests that suicidality reflects a network-level dysregulation involving inflammatory cytokines, oxidative stress, and kynurenine-pathway activation, though findings remain largely cross-sectional and preliminary [24,35,40].

#### 3.5.4. Clinical Implications

Although group-level cytokine alterations are commonly observed, their translation into clinical diagnostics remains challenging. Cytokine assays lack standardization and are susceptible to multiple biological and methodological confounders, including sampling factors and patient-related variables [13,40]. Therefore, cytokines should be viewed primarily as pathophysiological correlates of suicidality rather than as independent diagnostic biomarkers [13,38]. Future directions should include multi-marker panels integrating cytokines with hematological indices, neuroimaging, and digital phenotyping data to improve predictive accuracy and clinical utility [26,43,44].

## 4. Discussion

### 4.1. Interpretation of Main Findings

The main studies investigating inflammatory biomarkers in suicidal ideation and suicide attempts in major depressive disorder are summarized in Table 1. The present review indicates that suicidal ideation and suicide attempts in the course of major depressive disorder are frequently associated with signs of peripheral immune-inflammatory activation at the group level. Across diverse study designs and biomarker categories, suicidal individuals tend to exhibit higher levels of acute-phase proteins, elevated hematological inflammatory indices, and increased concentrations of proinflammatory cytokines compared with healthy controls and, in a subset of studies, with non-suicidal depressed patients.

Importantly, the magnitude of these differences is generally modest and characterized by substantial overlap between groups, underscoring that inflammatory biomarkers do not distinguish suicidal from non-suicidal individuals at the individual level. Rather than reflecting a suicide-specific biological signature, these markers appear to capture a state of heightened immune-inflammatory activity associated with more severe, acute, or biologically burdened depressive phenotypes.

Within this framework, the immune-inflammatory response system (IRS) and the compensatory immune-regulatory reflex system (CIRS) provide a useful interpretative model [35]. Suicidal ideation and suicide attempts are most frequently associated with increased activity of the IRS—manifested by elevated proinflammatory cytokines and acute-phase reactants—alongside insufficient or dysregulated counter-regulatory anti-inflammatory signaling. This imbalance may amplify downstream neurobiological processes relevant to mood dysregulation, impulsivity, and stress reactivity.

Hematological inflammatory indices derived from routine complete blood count represent a pragmatic reflection of this systemic immune activation. Elevated neutrophil-to-lymphocyte ratio, and to a lesser extent platelet-to-lymphocyte and monocyte-to-lymphocyte ratios, have been reported in suicidal subgroups across multiple studies. However, the absence of standardized sampling protocols and the predominance of cross-sectional designs preclude firm conclusions regarding temporal precedence or causality.

Taken together, the reviewed evidence supports an association between peripheral inflammatory activation and suicidal ideation or suicide attempts in major depressive disorder, while also highlighting the limitations of current data. The findings are best interpreted as group-level associations reflecting immune-inflammatory burden rather than as reliable individual-level predictors of suicidal behavior.

### 4.2. Clinical Implications and Limitations

The findings summarized in this review suggest that peripheral inflammatory biomarkers are frequently associated with suicidal ideation and suicide attempts in the context of major depressive disorder at the group level. However, their immediate translation into routine clinical practice remains limited. Importantly, none of the investigated biomarkers—whether acute-phase proteins, hematological indices, or cytokines—can be considered diagnostic or screening tools for suicidality.

Acute-phase proteins such as C-reactive protein and homocysteine are widely available and standardized in clinical laboratories, making them attractive candidates for large-scale observational studies. Nevertheless, their low specificity and strong susceptibility to metabolic, inflammatory, and lifestyle-related confounders substantially limit their clinical interpretability. Elevated levels may reflect a general inflammatory burden rather than suicide-specific risk.

Hematological inflammatory indices derived from routine complete blood count, including the neutrophil-to-lymphocyte ratio, platelet-to-lymphocyte ratio, and monocyte-to-lymphocyte ratio, offer practical advantages such as low cost, accessibility, and reproducibility. These indices may capture low-grade systemic inflammation relevant to depressive suicidality; however, they remain nonspecific and are characterized by substantial overlap between suicidal and non-suicidal individuals. At present, no validated reference ranges or cut-off values exist for their use in suicide risk assessment.

Cytokine profiling provides stronger mechanistic insight into immune–brain interactions and neuroinflammatory processes implicated in suicidality. Elevated proinflammatory cytokines and reduced anti-inflammatory signaling support the concept of immune-inflammatory imbalance; however, cytokine assays are limited by high biological variability, lack of assay standardization, circadian effects, medication exposure, and limited availability in routine clinical settings. Consequently, their current utility is largely restricted to research contexts.

Taken together, inflammatory biomarkers should be regarded as complementary biological information that may enhance understanding of suicidal vulnerability when interpreted alongside clinical assessment, rather than as standalone indicators of suicide risk. The practical advantages and limitations of the main biomarker groups discussed in this review are summarized in Table 2.

Several methodological limitations further constrain clinical translation. These include substantial heterogeneity in study design, variability in definitions of suicidal outcomes, cross-sectional sampling, and inadequate control for key confounding factors such as age, sex, body mass index, smoking status, comorbid substance use, and psychotropic medication. These limitations underscore the need for cautious interpretation of existing findings and highlight the importance of standardized, prospective, and multimodal approaches in future research. Although the primary focus of this review is suicidality in major depressive disorder, several high-quality meta-analyses include mixed affective populations; their findings are therefore interpreted cautiously as supportive, but not disorder-specific, evidence.

### 4.3. Methodological Considerations and Future Perspectives

The interpretation of inflammatory biomarkers in suicidal ideation and suicide attempts is constrained by several important methodological limitations that characterize the existing literature. First, most available studies employ cross-sectional designs, which preclude causal inferences and limit the ability to determine whether immune-inflammatory activation precedes, accompanies, or follows suicidal phenomena. Longitudinal studies with repeated biomarker measurements before and after suicidal crises are therefore critically needed.

Second, substantial heterogeneity exists in the definition and assessment of suicidal outcomes. Studies variably examine suicidal ideation, recent suicide attempts, lifetime history of attempts, or mixed endpoints, often without standardized operational criteria. This variability complicates cross-study comparisons and underscores the importance of adopting standardized nomenclature and outcome definitions in future research.

Third, methodological variability in biomarker assessment further limits comparability. Differences in biological sample type (serum vs. plasma), timing of sample collection relative to suicidal events, assay platforms, and analytical thresholds contribute to inconsistent findings. In addition, many studies lack adequate control for key confounding factors known to influence inflammatory markers, including age, sex, body mass index, smoking status, comorbid substance use, medical comorbidities, and psychotropic medication exposure.

Future research should prioritize large, prospective, multicenter cohort studies employing standardized sampling protocols and harmonized biomarker panels. Integrating inflammatory markers with complementary modalities—such as neuroimaging, genetic and epigenetic data, neurocognitive measures, and digital phenotyping—may enhance the ability to capture the complex, multidimensional nature of suicidal risk. In this context, machine learning-based approaches may facilitate the development of multivariable risk models that outperform single-marker strategies. Comorbid substance use and substance use disorders represent important and frequently undercontrolled confounders in studies of inflammatory biomarkers and suicidality, given their independent effects on immune activation and suicide risk.

Finally, a transdiagnostic perspective may prove valuable in disentangling diagnosis-specific versus shared inflammatory mechanisms of suicidality across mood and other psychiatric disorders. However, such approaches should be grounded in well-characterized clinical phenotypes and rigorous methodological frameworks to avoid overgeneralization. Collectively, these considerations highlight that inflammatory biomarkers currently represent promising research tools rather than clinically actionable indicators, and that their future utility will depend on methodological refinement, standardization, and integration within broader precision psychiatry paradigms. Major sources of heterogeneity affecting the interpretation of inflammatory biomarkers in suicidality are summarized in Table 3.

## 5. Summary

The accumulated evidence over the past decade provides converging evidence implicating immune-inflammatory dysregulation in the pathophysiology of suicidal behavior [13,29,40]. Across diverse study designs—ranging from cross-sectional case–control analyses to systematic reviews and meta-analyses—multiple peripheral inflammatory markers have been associated with increased suicidal behavior in patients with major depressive disorder and other affective illnesses.

Available evidence supports a conceptual model in which suicidal ideation and suicide attempts in major depressive disorder are associated with an imbalance between the immune-inflammatory response system (IRS) and the compensatory immune-regulatory reflex system (CIRS). Increased levels of proinflammatory markers (e.g., CRP, IL-6, TNF-α, IL-1β) alongside reduced or insufficient anti-inflammatory signaling in some clinical cohorts (e.g., IL-4, IL-10) may promote peripheral immune activation, kynurenine pathway dysregulation, and downstream neurotoxic processes. This imbalance is most frequently observed at the group level in suicidal ideation and attempts; data on suicide death remain limited and heterogeneous.

Among the evaluated biomarkers, C-reactive protein (CRP)—as highlighted in recent systematic analyses—has emerged as a readily accessible indicator of peripheral inflammatory activation in individuals exhibiting suicidal behavior [23]. In parallel, hematological indices derived from routine complete blood count, including NLR, MLR, PLR, as well as composite markers such as SII and NAR, capture low-grade systemic inflammation in an inexpensive and clinically accessible manner [12].

Meanwhile, cytokine profiling—particularly consistently elevated levels of pro-inflammatory markers such as IL-6, TNF-α and IL-1β—highlights the presence of peripheral immune activation in individuals with suicidality. These findings are consistent with evidence linking inflammatory dysregulation to neurobiological alterations relevant to suicidal behavior [13,26,40].

Taken together, these findings suggest that inflammation-related biomarkers could contribute objective biological information as an adjunct to clinical assessment of suicide risk [37,44]. However, several limitations restrict their immediate translation into clinical practice:

1. Heterogeneity of study designs—variability in inclusion criteria, timing of sample collection, and analytical methods undermines reproducibility [13].
2. Confounding factors—obesity, infection, medication use, and lifestyle variables can substantially influence inflammatory marker levels [29,40].
3. Lack of standardization—no universally accepted reference ranges or cut-off values for inflammatory indices exist in psychiatric populations [29].
4. Cross-sectional nature of evidence—most studies provide associative rather than causal data, precluding temporal inferences about inflammatory activation preceding suicidal acts [13].

At present, no validated reference ranges or clinically applicable cut-off values exist for inflammatory biomarkers in the assessment of suicidal ideation or suicide attempts. Reported changes are generally small to moderate at the group level and show substantial overlap between suicidal and non-suicidal individuals. Therefore, inflammatory biomarkers should be regarded as exploratory research tools and potential adjuncts to clinical assessment rather than diagnostic or screening markers. Future research should therefore focus on prospective, multicenter cohort studies employing standardized sampling, high-sensitivity immunoassays, and integrated multimodal approaches combining biological, neuroimaging, and digital phenotyping data [43,44]. Machine learning-based predictive models incorporating inflammatory biomarkers may help improve multivariable suicide risk stratification in research settings [43,44].

In conclusion, inflammatory biomarkers represent a promising but still exploratory diagnostic modality. Their greatest value likely lies in complementing—not replacing—clinical judgment [38,44]. To date, no clear cut-off values have been established for any of the markers, and data on their potential modulation by age, sex, or medication use remain limited. A more refined understanding of immune–brain interactions could ultimately open new avenues for personalized prevention and therapeutic strategies targeting inflammation in individuals at risk of suicide within a precision psychiatry framework [13,29,38,44].

## 6. Future Directions

Future research should prioritize large, multicenter, and longitudinal studies using standardized biomarker panels and harmonized sampling procedures to address current methodological heterogeneity. Establishing validated reference ranges, improving assay reproducibility, and integrating multimodal data, including clinical, biological, and digital domains (e.g., cytokines, hematological indices, metabolic markers, neuroimaging, and digital phenotyping), will be essential for determining the potential diagnostic and predictive utility of these biomarkers in suicide risk assessment. Such approaches may facilitate the development of biologically informed, individualized risk models within emerging precision psychiatry frameworks.

## Figures and Tables

**Table 1:** The main studies investigating inflammatory biomarkers in suicidal ideation and suicide attempts in major depressive disorder.

Author (Year)	Study Design	Population	Suicidal Outcome	Biomarker(s)	Sample Type	Main Findings	Key Limitations
Miola et al. (2021) [23]	Systematic review and meta-analysis	MDD and mixed psychiatric samples	SI/SA	CRP	Serum	Moderate elevation of CRP in suicidal individuals compared with non-suicidal individuals (psychiatric and/or healthy controls (SMD ~0.39)	High heterogeneity; confounding by BMI and somatic inflammation
Neupane et al. (2023) [13]	Systematic review and meta-analysis	MDD and affective disorders	SI/SA	Immune-related biomarkers (primarily cytokines)	Serum/plasma	Immune-inflammatory alterations associated with suicidal behavior at the group level	Group-level effects; limited individual-level prediction
Vasupanrajit et al. (2021) [24]	Systematic review and meta-analysis	Mood disorders	SI/SA	IL-6; TNF-α; IL-1β; oxidative markers	Serum/plasma	Activated immune-inflammatory and neurotoxic pathways in suicide attempts	Substantial heterogeneity; mixed diagnostic populations
Fernández-Sevillano et al. (2021) [16]	Cross-sectional clinical study	MDD patients	SI/SA	CRP; IL-6; TNF-α	Serum	Higher inflammatory markers in suicide attempters vs. non-attempters	Cross-sectional design; small sample
Susam et al. (2024) [17]	Cross-sectional study	MDD patients	SA	Homocysteine	Serum	Elevated homocysteine levels in suicide attempters	Small sample; nutritional confounders
Velasco et al. (2023) [14]	Systematic review	MDD patients	SI/SA	NLR; PLR; MLR	Whole blood	NLR generally higher in suicidal patients; mixed and inconsistent findings for PLR and MLR	Heterogeneous methods; lack of cut-off values
Kılıç et al. (2024) [12]	Case–control study	MDD patients	SA	NLR; PLR; MLR	Whole blood	Higher NLR in suicide attempters compared to non-attempters	Single-center; cross-sectional
Ninla-Aesong et al. (2024) [37]	Cross-sectional study	Mood disorders	SI/SA	SII; NLR	Whole blood	Elevated SII and NLR associated with suicide attempts	Exploratory indices; small sample
Yang et al. (2024) [25]	Cross-sectional clinical study	MDD patients	SI	IL-6; IL-10; TNF-α	Serum	Imbalance between pro- and anti-inflammatory cytokines in suicidal ideation	Cytokine variability; medication effects

**Table 2:** Biomarkers wit examples and analysis.

Biomarker Group	Examples	Main Advantages	Main Limitations	Clinical Interpretability
Acute-phase proteins	CRP; homocysteine	Widely available; standardized laboratory assays; inexpensive; routinely used in clinical practice	Low specificity; strong influence of metabolic and inflammatory confounders (BMI, infection, smoking); no validated cut-off values	Limited; may reflect general inflammatory burden rather than suicide-specific risk
Hematological inflammatory indices	NLR; PLR; MLR; SII; NAR	Derived from routine complete blood count; low cost; easily reproducible; suitable for large-scale screening studies	Nonspecific markers of systemic inflammation; lack of standardized reference ranges; substantial overlap between suicidal and non-suicidal individuals	Exploratory; potentially useful as adjunctive markers but not for standalone risk assessment
Cytokines	IL-6; TNF-α; IL-1β; IL-4; IL-10	Strong mechanistic relevance to neuroinflammation and immune–brain signaling; supported by meta-analytic evidence	High biological variability; assay-dependent results; limited availability in routine clinical settings; influenced by circadian rhythm and medication	Primarily research-oriented; limited immediate clinical applicability

**Table 3:** Heterogeneity of study.

Source of Heterogeneity	Description	Examples	Potential Impact on Findings
Clinical heterogeneity	Variation in suicidal outcomes and clinical state	Suicidal ideation vs. suicide attempts; acute vs. chronic depression; recent vs. lifetime attempts	Limits comparability across studies; may dilute effect sizes
Diagnostic heterogeneity	Differences in psychiatric diagnoses	MDD vs. mixed mood disorders; inclusion of bipolar or psychotic disorders	Obscures diagnosis-specific biomarker profiles
Methodological heterogeneity	Differences in study design and outcome assessment	Cross-sectional vs. cohort studies; variable definitions of suicidality	Precludes causal inference; inconsistent results
Biological sampling heterogeneity	Variability in biological materials and timing	Serum vs. plasma; fasting vs. non-fasting samples; timing relative to suicidal crisis	Affects biomarker concentrations and reproducibility
Analytical heterogeneity	Differences in laboratory assays and thresholds	High-sensitivity vs. standard CRP assays; different cytokine platforms	Leads to variability in reported levels and effect estimates
Demographic heterogeneity	Variation in population characteristics	Age; sex distribution; ethnic background	Modifies inflammatory responses and biomarker levels
Clinical confounders	Uncontrolled factors influencing inflammation	BMI; smoking; substance use; medical comorbidities; psychotropic medication	Introduces bias and reduces specificity of biomarkers
Statistical heterogeneity	Differences in analytical approaches	Variable covariate adjustment; multiple testing without correction	Inflates false-positive or inconsistent findings

## Data Availability

No new data were created or analyzed in this study.

## Abbreviations

The following abbreviations are used in this manuscript:

BMI	body mass index
CBC	complete blood count
CIRS	compensatory immune-regulatory reflex system
CRP	C-reactive protein
IDO	indoleamine-2,3-dioxygenase
IL	interleukin
IRS	immune-inflammatory response system
MLR	monocyte-to-lymphocyte ratio
MDD	major depressive disorder
NAR	neutrophil-to-albumin ratio
NLR	neutrophil-to-lymphocyte ratio
PLR	platelet-to-lymphocyte ratio
SA	suicide attempt
SI	suicidal ideation
SII	systemic immune-inflammation index
TNF-α	tumor necrosis factor alpha
